# Transient Horner’s Syndrome: An Unusual Complication of Pneumothorax Treatment

**DOI:** 10.7759/cureus.55033

**Published:** 2024-02-27

**Authors:** Priscila Fiallo, Hemangi Patel, Nithya G Devanathan, Sudeep Yadav, Alejandro Biglione

**Affiliations:** 1 Internal Medicine, Wellington Regional Medical Center, Wellington, USA; 2 Sports Medicine, Nova Southeastern University Dr. Kiran C. Patel College of Osteopathic Medicine, Fort Lauderdale, USA; 3 Medical School, Nova Southeastern University Dr. Kiran C. Patel College of Osteopathic Medicine, Davie, USA; 4 Medicine, University of Chicago Pritzker School of Medicine, Chicago, USA; 5 Internal Medicine, B. P. Koirala Institute of Health Sciences, Dharan, NPL

**Keywords:** thoracostomy tube, marijuana use, primary spontaneous pneumothorax, pneumothorax ptx, horner’s syndrome

## Abstract

Horner’s syndrome is a rare condition that results when there is an interruption of the sympathetic fibers that run from the stellate ganglion to the eye. The classic triad of Horner’s syndrome includes unilateral ptosis, miosis, and anhidrosis. Spontaneous pneumothorax is a rare condition that occurs when there is a sudden collapsed lung without any direct cause. A few cases have been reported of spontaneous pneumothorax associated with iatrogenic Horner’s syndrome. A chest thoracostomy is a procedure that can lead to iatrogenic Horner’s syndrome. Here, we present the case of a 25-year-old male with a left-sided spontaneous pneumothorax complicated by iatrogenic Horner’s syndrome secondary to chest thoracostomy.

## Introduction

Horner’s syndrome presents with unilateral ptosis, miosis, and anhidrosis on the affected portion of the face. The oculosympathetic pathway is damaged in this condition, resulting in these three hallmark symptoms. The first-order neurons are located in the hypothalamus and pass through the lateral brainstem [1]. The preganglionic sympathetic neurons then pass the pulmonary apex and travel to the stellate ganglion via the carotid sheath, reaching the superior cervical ganglion. Around 44% of cases involving Horner’s syndrome entail the preganglionic sympathetic neurons [1]. Thoracostomy, stellate ganglion block, and tumor excisions are procedures that can elicit symptoms.

In patients undergoing tube thoracostomy, the apices of the lung are a common site for tube insertion for the treatment of spontaneous pneumothorax. However, this can put the thin endothoracic fascia, which separates the pleura from the stellate ganglion, at risk of damage, leading to Horner’s syndrome. In roughly 1.2% of cases of thoracostomy tube placements, Horner’s syndrome can occur within 12 hours [2,3]. We present the case of a 25-year-old male who developed a transient Horner’s syndrome after chest thoracostomy for spontaneous pneumothorax.

## Case presentation

A 25-year-old male presented to the emergency department with the chief complaint of chest pain. The pain started abruptly while he was driving on the day of admission. He described the pain as a stabbing, constant, 10/10, diffuse pain. The patient denied any other symptoms at the time of anamnesis.

The initial vital signs included a heart rate of 113 beats per minute, blood pressure of 140/103 mmHg, respiratory rate of 22 respirations per minute, temperature of 97.9°F (36.6°C), and oxygen saturation of 97%. On physical examination, he was a thin and tall male who appeared to be in distress due to pain with diminished breath sounds on the left lung. It is worth mentioning that the patient disclosed undergoing extensive genetic studies for Marfan’s disease which were negative in the past.

**Figure 1:**
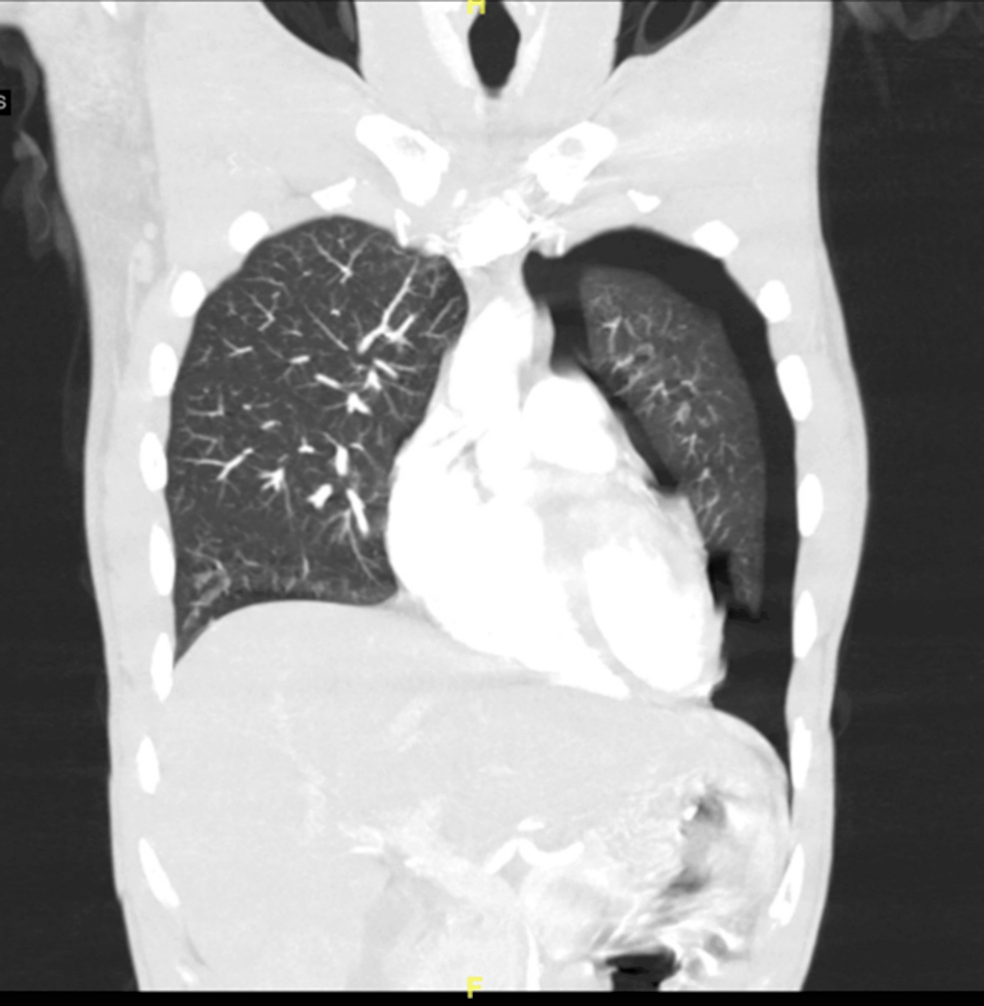
Computerized tomography with angiography of the thorax. Left-sided pneumothorax can be visualized.

The patient’s laboratory results included white blood cells (WBC) of 11.90 x 10^3^/µL, hemoglobin of 15 g/dL, and platelet count of 230 x 10^3^/µL. The patient underwent computerized tomography (CT) with angiography (Figure 1) which showed a left-sided pneumothorax without mediastinal side. A chest thoracostomy was placed on the left side, with chest radiography confirming positioning.

**Figure 2:**
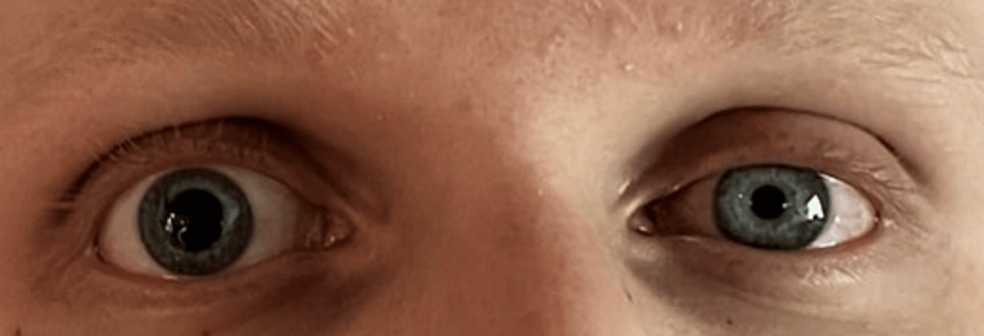
Photograph of Horner’s syndrome. The image reveals marked anisocoria, miosis, and partial ptosis of the left eye.

**Figure 3:**
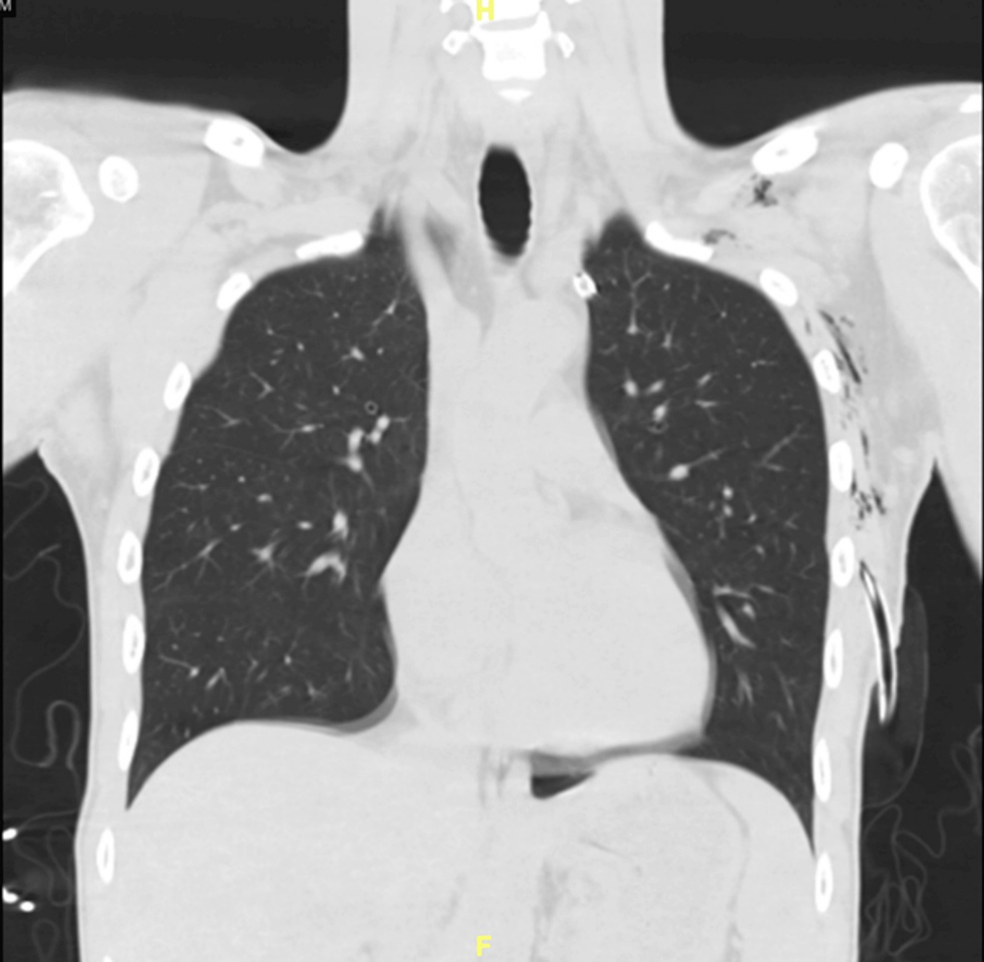
Computerized tomography of the thorax with chest thoracostomy. Left-side chest thoracostomy with the appearance of the abutted sympathetic nerve.

**Figure 4:**
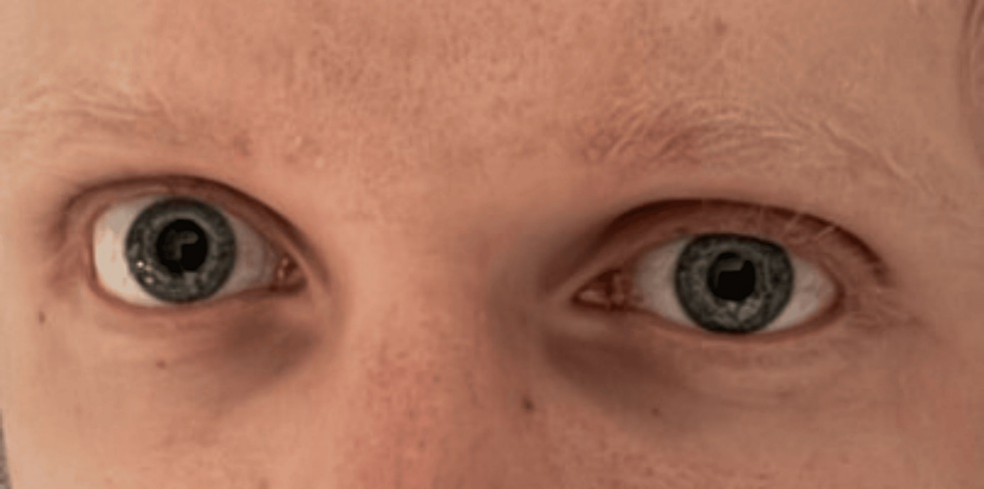
Resolution of Horner’s syndrome. Chest tube repositioning resulted in the resolution of symptoms.

On day five, the patient developed anisocoria, with partial ptosis, and miosis of the left eye (Figure 2). At the time of the finding, the chest tube was suspected to be abutting the sympathetic nerve, causing Horner’s syndrome. A CT of the thorax confirmed the impinged nerve in the topography of the stellate ganglion (Figure 3). The thoracic surgeon on the case was notified, and after repositioning the chest tube, the symptoms improved instantaneously (Figure 4).

Finally, the patient underwent chemical pleurodesis, as indicated, after more than five days without resolution of the pneumothorax. The patient was discharged home with indications to follow up with his primary care physician and the thoracic surgeon.

## Discussion

Horner’s syndrome is a condition caused by disruption to the oculosympathetic pathway, resulting in unilateral ptosis, miosis, and anhidrosis. The damage to the pathway can occur anywhere between the hypothalamus, brainstem, stellate ganglion, and the trigeminal nerve. In some cases, irritation to the stellate ganglion located near the lung apices occurs and leads to reversible damage [4]. The findings in the case described suggest an interruption of the neural conduction pathway without permanent structural damage. The cause of Horner’s syndrome in our patient may be due to the direct pressure from the tip of the tube inserted into the apex of the chest.

There is minimal literature on the exact mechanism and whether the migration of the tube during the inpatient stay can be avoided. The other explanations for the delayed symptoms of Horner’s syndrome include inflammation, fibrosis, and local hematoma formation. In this case, no acute new findings contributed to Horner’s syndrome, making migration of the tube after the clamping of the thoracostomy tube the likely cause. Once the tube was repositioned, the symptoms resolved.

Routine chest radiography does not visualize the chest tube to sympathetic chain relationship. Still, it is an important step as it can rule out many of the other causes of Horner’s syndrome after chest tube placement. Further, CT of the chest with contrast can be done if symptoms do not resolve after chest tube repositioning because it can diagnose more emergent and dangerous diagnoses such as vascular dissection or pseudoaneurysm formation [4].

## Conclusions

Horner’s syndrome is a rare condition involving the impingement of the stellate ganglion. In the case discussed, iatrogenic Horner’s syndrome occurred secondary to left-sided chest tube thoracostomy in a 25-year-old male with a spontaneous pneumothorax. The chest tube thoracostomy caused an impingement of the stellate ganglion in the left lung apices. This exemplifies the importance of recognizing the signs and symptoms of Horner’s syndrome, proper management, and daily imaging to confirm proper tube placement. Horner’s syndrome occurs in a very minute population and, if not treated promptly, can cause permanent irreversible damage. Thus, properly managing patients is important to avoid long-term complications.
